# Position statement of the Brazilian Society of Nephrology on the refusal and discontinuation of dialysis

**DOI:** 10.1590/2175-8239-JBN-2025-0057en

**Published:** 2025-08-15

**Authors:** Dirceu Reis da Silva, Fernanda Salomão Gorayeb-Polacchini, Ana Flávia Moura, Cibele Isaac Saad Rodrigues, Maurício Younes-Ibrahim, Eduardo Rocha, Marina Aline Occhiena de Oliveira Neiva, Pedro Túlio Rocha, Patrícia Ferreira Abreu, José A. Moura-Neto

**Affiliations:** 1 Sociedade Brasileira de Nefrologia, São Paulo, SP, Brazil.; 2 Hospital de Clínicas de Porto Alegre, Porto Alegre, RS, Brazil.; 3 Instituto de Doenças Renais, Porto Alegre, RS, Brazil.; 4 Hospital de Base da Faculdade de Medicina de São José do Rio Preto, São José do Rio Preto, SP, Brazil.; 5 Escola Bahiana de Medicina e Saúde Pública, Salvador, BA, Brazil.; 6 Pontifícia Universidade Católica, Faculdade de Ciências Médicas e da Saúde, Sorocaba, SP, Brazil.; 7 Hospital Santa Lucinda, Sorocaba, SP, Brazil.; 8 Universidade do Estado do Rio de Janeiro, Rio de Janeiro, RJ, Brazil.; 9 Pontifícia Universidade Católica do Rio de Janeiro, Rio de Janeiro, RJ, Brazil.; 10 Renalle Consultoria e Serviços Médicos, Petrópolis, RJ, Brazil.; 11 Universidade Federal do Rio de Janeiro, Rio de Janeiro, RJ, Brazil.; 12 Hospital Santa Casa de Misericórdia de Goiânia, Goiânia, GO, Brazil.; 13 Hospital do Rim, Goiânia, GO, Brazil.; 14 Hospital São Lucas Copacabana, Rio de Janeiro, RJ, Brazil.; 15 Universidade Federal de São Paulo, São Paulo, SP, Brazil.

**Keywords:** Dialysis, Acute Kidney Injury, Advance Directives, Withholding Treatment, Medical Futility, Palliative Care, Bioethics

## Abstract

Renal failure is considered a life-limiting CONDITION that often requires Renal Replacement Therapy, such as dialysis or kidney transplantation. Dialysis can effectively relieve symptoms and prolong life, but its withdrawal results in severe complications and death. The decision to discontinue or refuse to dialysis must be made collaboratively by the patient, family, and healthcare team, considering the clinical condition, life expectancy, symptom burden, and individual preferences. This decision, involving clinical, bioethical, and legal aspects, is complex and requires a collective understanding of the process. Withdrawal to dialysis presents a challenge for nephrologists and the healthcare team due to the lack of clear guidelines, which can compromis the safety of the process and the patient’s dignity. In this position statement, the Brazilian Society of Nephrology recommends a process for dialysis withdrawal or refusal, including identifying eligible patients, applying prognostic assessment tools, shared decision-making, advance care planning, and offering dialysis alternatives. The decision must be consensual, allowing adequate time for reflection, and healthcare services must provide comprehensive management of physical, psychological, social, and spiritual symptoms, as well as end-of-life care. Proper documentation in medical records is essential to ensure process transparency. Therefore, refusal or withdrawal to dialysis should be an informed decision that respects individual autonomy and balances clinical, bioethical, spiritual, and legal considerations.

## Introduction

Particularly in its more advanced stages, renal failure may be considered a potentially life-limiting condition, thus requiring the adoption of Renal Replacement Therapy (RRT), either through one of the dialysis modalities or kidney transplantation^1^. Within the scope of this discussion, we comprehensively address scenarios involving chronic kidney disease (CKD) and acute kidney injury (AKI), referring to dialysis as a general concept that encompasses its various modalities, such as peritoneal dialysis (PD), hemodialysis (HD), and hemodiafiltration (HDF).

Dialysis is widely recognized as an effective treatment for alleviating symptoms resulting from advanced renal failure, and for prolonging the lives of these patients^1^. As a life-sustaining therapy, its withdrawal or refusal inevitably leads to serious complications such as hypervolemia, hyperkalemia, or uremia, tending to fatal outcome in approximately 7.8 days^2^.

**Figure 1:**
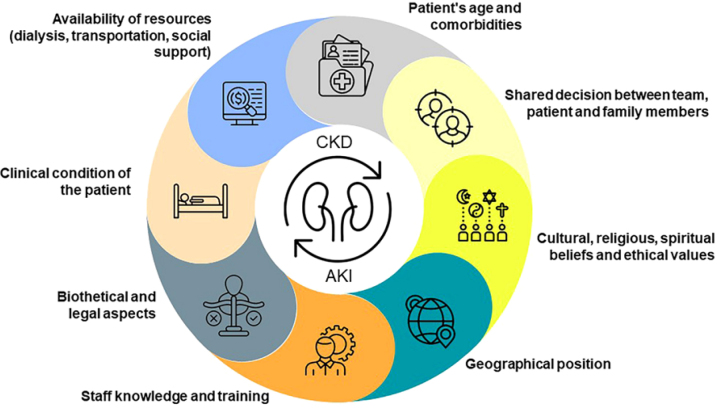
Aspects that may interfere with the process of discontinuing or refusing dialysis.

Therefore, considering the patient’s clinical condition, quality of life, and preferences, the decision for dialysis withdrawal should be made through a shared decision-making process involving the patient, their family, and the healthcare team^3^. This is a complex judgment (see Figure 1), which requires a collective process of understanding and construction. In this context, dialysis withdrawal poses a challenge for nephrologists and the care team, resulting in limitations in the consistency of deliberation and the availability of end-of-life care^4^.

Abbreviations – CKD: Chronic Kidney Disease; AKI: Acute Kidney Injury.

In Brazil, adequate training for healthcare professionals and the development of national guidelines are still needed to ensure greater certainty in defining procedures, preserving patient dignity, protecting the emotional well-being of family members and healthcare professionals, and reducing legal risk^5^.

The objectives of this document are: 1) to contribute to improving the quality of the dying process for patients with advanced kidney disease; 2) to mitigate the adverse effects of this painful experience on families and to support the emotional well-being of healthcare professionals in this context; and 3) to serve as a foundation for the development of protocols and tools intended for healthcare professionals, hospital administrators, and healthcare policymakers, in accordance with local circumstances.

## Discussion

### Definition

Despite its growing acceptance in developed countries, the decision to withhold dialysis in patients with renal failure still lacks a clear and consistent definition across studies^4,5,6,7^. In the present position statement, we address its two possible forms: refusal and discontinuation. Refusal, which is more common, refers to the decision not to initiate dialysis treatment in patients with advanced-stage kidney disease, for whom dialysis would be traditionally and technically indicated as a life-sustaining intervention^7^. Conversely, discontinuation consists of the decision to discontinue dialysis that has already been initiated and is necessary for life support^6–7^, or of the confirmation of death adequately attributed to renal failure in a patient who has discontinued dialysis^4^.

In this document, we adopt the term “dialysis withdrawal” as a broad concept that encompasses two distinct possibilities: refusal and discontinuation of dialysis treatment.

### Epidemiology

Dialysis withdrawal has been increasingly common among patients with renal failure, showing wide geographical variability. This variation is related to patient profile, cultural, religious, and spiritual beliefs, ethical values, and the greater or lesser availability of RRT^4,8^.

In a systematic review that evaluated the survival of patients with advanced CKD who had been referred to RRT but opted for conservative treatment (dialysis refusal) , this decision resulted in a median survival of at least six months, ranging from 6.3 to 23.4 months^9^. In another systematic review and meta-analysis, analyzing elderly patients with advanced CKD, one-year survival was similar between those receiving dialysis and those who underwent conservative treatment, although no comparisons of quality of life were performed^10^.

In a DOPPS study involving 259,343 patients, 12% of deaths were attributed to dialysis discontinuation. In these cases, median survival time after treatment discontinuation was seven days (interquartile range, 5 to 9 days), with only 4.4% dying after more than 30 days^11^. Discontinuation of dialysis tends to be more frequent among the elderly^12^, female patients^13^, residents of rural areas^13^, patients with degenerative diseases^12^ or disabilities^13^, patients on intermittent PD^12^, or those who are institutionalized^12^.

In countries with high *per capita* income, dialysis discontinuation has become one of the leading causes of death among patients undergoing dialysis therapy^11,12,13,14,15,16,17,18^. Despite the scarcity of Brazilian data in the literature, it is assumed that these rates are lower than those observed in developed countries due to social, cultural, religious, and economic reasons^5^.

### Patients with Advanced CKD Undergoing Conservative Treatment or in Dialysis Clinics

While kidney transplantation has eligibility criteria and contraindications that prevent its universal availability to patients with advanced CKD^19^, dialysis is virtually applicable to any individual requiring RRT. In addition, ongoing demographic changes in the country, particularly population aging and an increase in people with chronic comorbidities, largely explain the rising prevalence of advanced CKD cases^20^. Therefore, it is crucial to discuss the prognosis with advanced CKD patients and their families, considering their medical conditions, possible therapeutic options (RRT or conservative treatment) – with their respective advantages and disadvantages - as well as the need for Advance Care Planning (ACP), so as to provide comfort, peace, and dignity at the end of life^8,21^. The steps for implementing ACP are outlined in Chart 1.

**Chart 1:** Stages of advance care planning (ACP) ^22^

Steps	Objectives
Preparation	– Confirm the details of the diagnosis, prognosis, and available treatment options prior to the conversation with the patient.
Permission/check autonomy	– Initially assess whether the patient is capable of making the decision independently and whether the presence of family members is necessary. – The patient, once deemed competent, should be asked whether they wish to make the decision themselves or share it with a family member or trusted person. – Patients with uncontrolled symptoms should only have this conversation after symptoms have improved.
Identification of the patient's perception of the disease, their values, and wishes	– Through active listening, restore the patient's and their family's understanding of the diagnosis, prognosis, and usual treatment options.
Discuss possible dilemmas and care preferences	– Explain the risks and benefits of the different treatment options. – Provide information on invasive and artificial life-sustaining measures.
Decision making and care plan	– Develop a care plan, aligning the available treatment options with the patient's preferences and priorities.
Document decisions	– Always document decisions in the medical record so that the entire care team has access to what was discussed and agreed upon. – Encourage the patient to write their Advance Directive (AD)*.
Identify a patient's healthcare representative	– Encourage the patient to designate a trusted person to represent them in situations where they are unable to communicate or have limited autonomy. – If the patient has not designated a representative, it is advisable to encourage them to share their preferences with close and trusted individuals to ensure that their wishes are respected.
Review the ACP	– Validate the content of the conversation and offer patients the opportunity to review their care plan at any time.

Abbreviations – ACP: Advance Care Planning.

Notes – *Preparation of an AD in the form of a living will and/or durable power of attorney. See details under (bio)ethical aspects.

The main reasons for discontinuing dialysis in this population include clinical factors (debilitating chronic comorbidities, disability, frailty, acute complications, and access difficulties) and psychosocial factors (mental health burden, lack of social support, and logistical reasons)^2,4,13^.

In order to reduce treatment burden and optimize quality of life, patients should be offered alternatives to conventional dialysis regimens^3–4,21^ (incremental dialysis^22,23,24,25,26^, palliative dialysis^27–28^ or time-limited trial of dialysis^29,30,31^), as detailed in Chart 2 ^22–31^.

**Chart 2:** Dialysis alternatives (hemodialysis or peritoneal dialysis) in patients who wish to or are candidates for dialysis discontinuation or refusal

Dialysis Alternatives	Type of kidney disease	How to do it	Objective	Follow-up
Incremental dialysis^22–26^	CKD	– Patients with RRF; – Usually 2 to 3 hours, twice a week.	– Preserving RRF, lower costs and hospitalization rates, better quality of life, and treatment adherence.	– Evaluate loss of RRF; – Adjust, if necessary, time or frequency according to the patient's clinical and laboratory status.
Palliative dialysis^27,28^	CKD or AKI	– Patients with limited life expectancy or severe comorbidities; – Dialysis or UF, only in case of symptoms.	– Comfort, improved quality of life, symptom management.	– Patient follow-up with a focus on symptom management.
Limited-time dialysis^29–31^	CKD or AKI	– Limited time agreed with patient and family members; – Establish temporary treatment goals.	– Time-limited treatment due to prognostic uncertainty, unresolved conflicts, potentially reversible causes.	– Patient assessment at the end of the period, according to the proposed objectives; – If necessary, reassess the management strategy during the period (change of decision, clinical condition, or prognosis).

Abbreviations – HD: Hemodialysis; PD: Peritoneal Dialysis; CKD: Chronic Kidney Disease; AKI: Acute Kidney Injury; RRF: Residual Renal Function; UF: Ultrafiltration.

Although dialysis discontinuation is common in high-income countries, the clinical approaches currently adopted in dialysis units often fail to meet the needs of patients at the end of life. In a Canadian study conducted in dialysis units, patients reported little knowledge about the course of their disease and about palliative care options; among them, 61% expressed regret over their decision to initiate dialysis, 27% said they would prefer to die in a hospital, and less than 10% reported having had the opportunity to discuss end-of-life care issues with their nephrologist in the past 12 months^32^. Therefore, dialysis centers need to develop their teams and disseminate written guidelines on how and when to offer dialysis discontinuation, and on how patients should proceed under comprehensive health care^8^. Furthermore, it is essential to incorporate palliative care training for resident physicians and nephrology interns, emphasizing symptom management and the implementation of ACP^33^.

For dialysis patients approaching the end of life, it is recommended to discuss appropriate end-of-life care and the potential discontinuation of dialysis through ACP. On the other hand, when evaluating the decision to discontinue treatment in patients with an uncertain life expectancy, potential treatable or manageable factors should be investigated, such as depression, anxiety, and pain; dissatisfaction or discomfort with dialysis; inadequate social support; and concerns about becoming a burden to loved ones^4,21^.

In the shared decision-making process of not initiating (refusal) or discontinuing dialysis for CKD patients, comprehensive clinical and multidisciplinary care should be offered, with a holistic, patient-centered approach that includes active symptom management as well as psychological, social, family, and spiritual support^21^.

### Specific Aspects of Dialysis Refusal in Patients with AKI or in The Intensive Care Unit (ICU)

AKI is a frequent event, affecting up to 82.5% of patients in ICU beds^34^, with mortality rates inversely proportional to the staging proposed by the Kidney Disease: Improving Global Outcomes (KDIGO)^34,35^. It is a clinical syndrome with variable phenotypes that may affect patients of all age groups, being more frequent among the elderly population^36^.

Decision-making regarding non-initiation (refusal) or discontinuation of RRT in AKI patients may be more complex than in CKD patients due to the following factors^37^: (a) potential for renal function recovery; (b) even in severe cases, acute illness may be perceived by both patients and physicians as reversible^38^; (c) critically ill patients are often unable to make medical decisions; (d) few critically ill patients have ACP and/or AD, and even when they do, these tools do not seem to assist in decision-making by their surrogates^39^. The clinical contexts of patients eligible for dialysis withdrawal in cases of AKI^37,39^ do not differ essentially from those observed in CKD patients, as described in Chart 3.

**Chart 3:** Patients who are potential candidates for dialysis refusal or discontinuation

1.	Patients with preserved decision-making capacity, fully informed, who voluntarily choose to withdraw from dialysis^48^
2.	Patients who are incapable of making decisions (due to clinical, neurological, or psychiatric causes) and who have documented ACP^33,53^, or whose duly appointed legal representatives choose to withdraw from dialysis^48^
3.	Patients with irreversible and profound neurological damage, with no signs of consciousness, sensation, intentional behavior, or awareness of self or surroundings^48^
4.	Patients with advanced CKD of non-renal origin or whose medical condition prevents dialysis from being technically feasible^48^
5.	Patients with advanced CKD not on dialysis who are at high risk of death within the next year^32^ (consider using a combined 6-month mortality prediction tool^54^ or the “Surprise Question” (‘Would you be surprised if this patient died within the next 12 months?’)^55^, and symptom assessment tools, such as the Edmonton Symptom Assessment Scale^56^)
6.	Patients undergoing dialysis to sustain life in the context of comorbid conditions in which quality of life and experience of living are inadequate and undesirable, or in which a fatal outcome is assured (e.g., a patient with advanced cancer without therapeutic options, or sepsis with multiple organ failure)^57^
7.	Patients for whom the effort required to implement dialysis, as well as its associated complications, outweigh the potential benefits of prolonging life (e.g., patients with progressive frailty who are becoming bedridden, or those with severe cognitive impairment)^57^

Abbreviations – ACP: Advance Care Planning; CKD: Chronic Kidney Disease.

Survival prediction scores for critically ill patients may assist in the decision-making process regarding dialysis withdrawal^40,41,42,43,44^, especially in elderly patients > 75 years^44^. Using specific tools, it is possible to establish objective criteria translated into indices that reveal a high degree of comorbidity (e.g., Modified Charlson Comorbidity Index >8^45^) or functional impairment (e.g., AKPS ≤ 40%^46^).

In critically ill patients with AKI associated with multiple organ failure and/or resulting from other uncontrolled comorbidities, dialysis withdrawal is considered when dialysis methods no longer play a therapeutic role and only serve to postpone the final outcome. In these patients, the decision not to initiate or to discontinue RRT, with the aim of providing proportionate care, is one of the challenges of hospital nephrology practice, even though the availability of new technologies allows RRT to be offered to most patients^47^. Given the complexity of this issue, there is no single approach suitable for all cases, and it is essential that decisions always be made on an individual basis^37,48^.

Additionally, discussing long-term prognosis with patients or their families is important to ensure that their wishes are respected. Data from patients with prolonged ICU stays (2 weeks) showed that only 38% had discussed prognosis with their physicians, and 47% of those who preferred a palliative approach believed they were receiving treatment contrary to their wishes^49^. Offering time-limited trial of dialysis^29,30^ or palliative dialysis^27^ may be a clinically and ethically appropriate option^30^ for AKI patients, as summarized in Chart 2.

Once the decision to limit invasive measures has been made, it is important to emphasize that discontinuation of life support may occur gradually. Discontinuation of dialysis is likely to be one of the first measures implemented, while discontinuation of nutrition, hydration, and mechanical ventilation tends to be among the last interventions withdrawn^50^. Follow-up by a palliative care team should be considered to assist in managing difficult-to-control symptoms, as well as in resolving potential conflicts among patients, family members, and healthcare professionals. Families should be aware of the possibility that patients may recover from AKI. Transfer to a non-ICU ward should be considered, depending on the patient’s preferences, their family’s coping ability, and the availability of trained end-of-life care personnel^37^.

Given the complexity of the issue, no level of evidence was sufficient to allow formal recommendations on different aspects^51^. With the increasing development of artificial intelligence tools, it would not be surprising if, in the short term, the validation of new algorithmic models were to categorically assist in decisions related to end-of-life care in critically ill patients^52^.

### Patients Eligible for the Dialysis Withdrawal Process

Different guidelines and recommendations^8,32,48^ have been published to guide situations in which patients are potentially eligible for dialysis withdrawal, as compiled in Chart 3 ^33,48,53,54,55,56,57^.

Among the various guidelines, the Renal Physicians Association (RPA) and the American Society of Nephrology (ASN) convened representatives from several organizations in the dialysis community – kidney patients, family members, internists, bioethicists, and public policy experts – and published the Clinical Practice Guideline on Shared Decision-Making in the Appropriate Initiation of and Withdrawal from Dialysis. This guideline establishes nine recommendations on dialysis withdrawal, including patients who are candidates for the process^48^.

In turn, KDIGO published that dialysis withdrawal is both ethically and clinically acceptable, and that potentially treatable factors that may contribute to this decision (such as depression and other mental disorders) or other symptoms (such as pain, pruritus, restless legs syndrome, sleep disorders, and fatigue) should always be addressed, in addition to considering reversible social factors^8^.

Withdrawal from dialysis poses a particular challenge in patients with dementia, as these individuals are unable to make their own decisions. A study using data from seven countries recognized that dementia is associated with an increased risk of death and a twofold higher likelihood of discontinuing dialysis, reinforcing the importance of adequate and regular medical screening for cognitive impairment in patients^58^.

### (BIO)Ethical and Legal Aspects Surrounding Dialysis Refusal or Discontinuation

- “*The good physician treats the disease; the great physician treats the patient who has the disease*.” – Sir William Osler

With advances in RRTs, patient survival has significantly increased, particularly among those with CKD^59^. However, the mere availability of advanced technology and well-trained multidisciplinary teams is not sufficient; it is essential that the patient remains the focus of our attention and care^60^. Thus, when considering the individual from a biopsychosocial perspective, grounded in principle-based ethics, it is crucial to consider the principles of beneficence (doing good) and nonmaleficence (doing no harm), not only in relation to patients, but also to their support network—which includes family members, caregivers, and the multidisciplinary team^61^.

Equally fundamental principles in the humanized approach to patients requiring RRT include the autonomy and protagonism of individuals, shared responsibility between healthcare professionals and patients, recognition of their vulnerabilities, the establishment of supportive bonds, and collective participation in the decision-making process^62^.

Currently, there is an arsenal of tools for maintaining vital functions, offering healthcare professionals the possibility of postponing the natural dying process for extended periods. However, it is worth asking: “What is the impact of this new reality on patients’ quality of life?”. The thanatological compulsion to fight death and the obsession with maintaining biological life at all costs have led to the concept of therapeutic obstinacy, or dysthanasia, which contradicts the principles of human dignity in the face of the limits of medicine and science^63,64^. This type of approach, which prioritizes quantity over quality of life, should be considered poor clinical practice and constitutes an unethical stance, as outlined as a violation in the Code of Medical Ethics^65^.

Since 2006, the Brazilian Federal Council of Medicine (CFM), through Resolution No. 1,805/2006, has allowed physicians to refrain from implementing and to discontinue treatments that prolong the life of patients in the terminal stage of severe and incurable illnesses, provided that the patient’s wishes or those of their legal representative are respected^66^. The main justifications provided by the CFM for this resolution are supported by Articles 1 and 5 of the Brazilian Federal Constitution, which establish the principle of human dignity as one of the country’s fundamental pillars and prohibit subjecting any individual to torture or to inhuman or degrading treatment. This resolution is in line with the position of international entities such as the World Medical Association, the Council of Europe, the European Court of Human Rights (ECHR), and the United Nations Educational, Scientific and Cultural Organization (UNESCO).

We still lack definitive answers as to what may occur between “doing everything” and “doing nothing.” More or less invasive procedures must respect the individuality and personal history of each patient, guided by the bioethical principles of autonomy, justice, beneficence, and nonmaleficence, with a view to comfort and quality of life. The goal should always be to care for patients in a manner consistent with their values at a time of unparalleled vulnerability, when they are rarely able to speak for themselves^67^. Ensuring that the end of life occurs at the right time, with dignity, care, and as much comfort as possible is referred to as orthothanasia (“good death”)^65,68^.

A more recent concept is that of calothanasia or kalothanasia, which, although still not widely disseminated, is more comprehensive than ortho­thanasia. It refers to the revival of death, the rational use of therapeutic resources to manage pain and suffering, thus avoiding unnecessary prolongation of the dying process. This concept is grounded in the hospice movement, which is based on respectful care for the beliefs and values of the patient and their family^69^.

It should also be noted that euthanasia – understood as death by mercy or compassion – is considered a crime under Brazilian law. This practice presupposes an “action” that causes death, whether actively or passively, the latter being constituted by negligence^65,68^.

In addition, it is essential to recognize that discontinuing and not implementing life-sustaining interventions in the context of a terminal illness differ from the concept of euthanasia. The International Association for Hospice and Palliative Care (IAHPC) and the European Association for Palliative Care (EAPC) have issued statements on the matter of euthanasia^70,71^, defining it as the action of a physician administering medication to a patient with the intention of causing death, based on a voluntary request made by the patient while fully capable of making decisions.

The humanitarian crisis in dialysis, denounced by the Brazilian Society of Nephrology (BSN), fits perfectly into the most recent definition of death: mistanasia^68^, where “mis” is a prefix meaning “unfortunate”. This is the term used to refer to the death of individuals, often socially marginalized, without any healthcare or with inadequate medical assistance. The late - or absent - diagnosis of CKD, the shortage of dialysis units to meet the demand of CKD and AKI patients, and the lack of resources to provide RRT support during the COVID-19 pandemic are examples of this type of death in the context of Nephrology^72,73,74^.

Advance Directives (AD) are documents that express the wishes previously and expressly stated by the patient regarding the care and treatment they wish to receive or not receive when they are no longer able to express their will freely and independently (CFM Resolution 1995/2012, Art. 1)^75^. ADs may be formalized through two distinct instruments: a living will and a durable power of attorney. Both may coexist and are intended to be used when the patient is unable to express themselves freely and consciously. A durable power of attorney allows the patient to appoint one or more representatives to be consulted in the event of temporary or permanent incapacity to make decisions regarding treatment or procedures. This instrument may be used when the patient has not previously expressed their wishes or in situations involving unforeseen circumstances (gaps), in which case the legal representative must decide, always based on the patient’s presumed will^76^.

The other type of AD, in addition to the durable power of attorney, is the living will. This is a legal document that emerged following the model of “living will” established in the US in the 1960s. It allows patients to state their preferences regarding the treatments they wish to authorize if they find themselves in a terminal condition, including the possibility of withholding extraordinary and futile procedures^75,76^.

According to CFM Resolution 1995/2012, ADs may be recorded by the physician in the patient’s medical record, provided that the patient or their representative authorizes it^75^. Witness signatures are not required, since physicians, by virtue of their professional status, hold public faith, and their acts carry legal and judicial validity. If desired, the individual may register their AD at a notary office, with witnesses, although this is not mandatory^75,76^.

Nephrologists are often confronted with issues closely related to the life and death of patients, both in the context of CKD and AKI, and are required to make distressing decisions. These decisions are best implemented when shared with members of hospital Bioethics Committees, with multidisciplinary teams at the dialysis centers, and especially with patients themselves and their families when the course of care includes palliative care, orthothanasia, and calothanasia. For nephrologists, keeping bioethical principles in mind helps in not recommending or even withdrawing dialysis when this represents the best course of action for the patient, always with respect for their autonomy^77^.

The decision to withhold or not initiate dialysis in CKD patients should not be made abruptly and must involve a documented process with informed consent signed by all parties involved^78^. This is always a shared decision between the responsible healthcare team (physicians and other professionals), the family, and, whenever possible, the patient themselves, and may include, if necessary, consultation with specialists in (bio)ethics and/or legislation. These situations must also ensure care, either by the nephrology team itself or through an experienced and efficient palliative care service^79^.

The position statement published by the Bioethics Committee of the National Academy of Palliative Care includes the following guidelines: (1) from a bioethical standpoint, there is no effective distinction between discontinuing and refusing life-sustaining interventions; (2) it is unethical to initiate or maintain life-sustaining measures in contexts where these are inconsistent with the values and goals of care defined in collaboration with patients and their families; (3) futile treatments – those with no possibility of achieving even their intended physiological purpose – should not be initiated or maintained, even at the request of the patient or their representatives; (4) with regard to life-prolonging treatments, their continuation may be inappropriate when their ethical basis is in question, and their refusal necessarily requires agreement on therapeutic goals, considering the patient’s values and perspectives^80^.

Offering alternatives to dialysis that promote a better quality of life is a duty of healthcare professionals. In addition, it is essential to avoid exposing patients to situations that may cause additional suffering, considering the balance between benefit and harm, between right and wrong. Even in the face of end-of-life situations, it is imperative to ensure that this process occurs with dignity and respect for the human condition.

### Palliative Care in Nephrology

The World Health Organization (WHO) defines palliative care as a comprehensive approach aimed at improving the quality of life of patients and their families, working together to address the challenges associated with life-threatening illnesses^81^, such as AKI or advanced CKD.

Effective management of suffering, distress, physical, emotional, social, and spiritual symptoms, and grief should be provided^82,83,84^. Palliative care aims to provide satisfaction to patients and their families, alleviate the pain of loss, reduce unnecessary costs, and, in some very specific situations, prolong life. These services can be provided in different settings, including outpatient clinics, hospitals, specialized centers, and at the home environment^82–84^.

**Figure 2:**
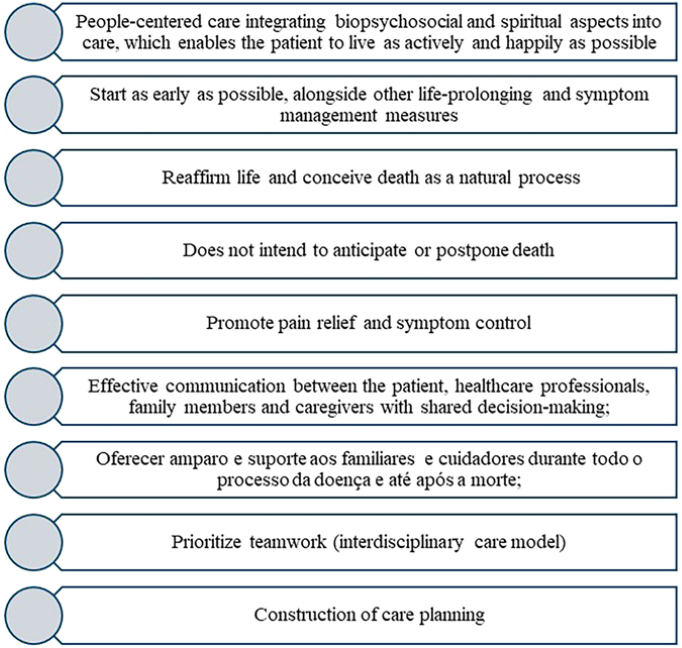
Pillars of Palliative Care.

The pillars of palliative care are described in Figure 2 ^82–84^.

Source – Adapted from references^82,83,84^.

**Figure 3:**
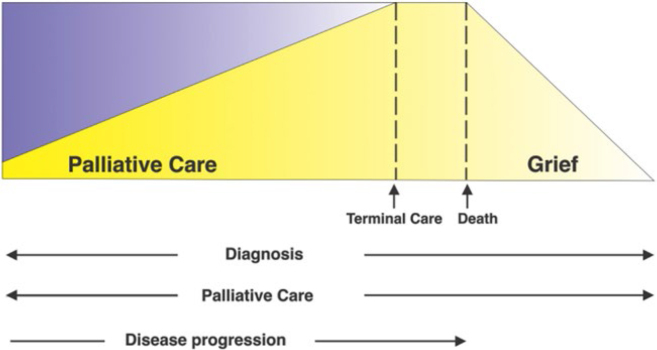
Potential Role of Palliative Care for Patients in Relation to the Stage of Illness. The overlap between curative and palliative care varies according to the clinical condition.

Over time, the focus and goals of care gradually shift from an emphasis on disease-modifying treatments to interventions with exclusively palliative intent. The benefits of palliative care increase as the symptom burden increases and death emerges as individual suffering, along a *continuum* in which disease-modifying treatments lose their effectiveness and applicability, extending to the active dying phase and the grieving of loved ones^85^. The potential role of palliative care in relation to the stage of the disease is depicted in Figure 3.

Source – Adapted from the Permanent Commission on Palliative Care of the Brazilian Society of Geriatrics and Gerontology. *Vamos falar de Cuidados Paliativos*, 2014, available at: https://sbgg.org.br/wp-content/uploads/2014/11/vamos-falar-de-cuidados-paliativos-vers--o-online.pdf.^85^

The lines are shown as straight lines for educational purposes only, as the intensity of each of the treatments offered simultaneously varies according to the needs and priorities of the patient and their family.

The annual mortality rate for dialysis patients is 16.2%^20^, reaching 38% among those aged 75 years or older and exceeding 50% in frail elderly patients^32^. Furthermore, the dialysis trajectory is characterized by slow deterioration, punctuated by abrupt and only partially reversible acute events, typical of organ failure^86^. Therefore, when planning individualized care for patients with AKI or CKD, the following strategies should be considered:

- ***Not initiating dialysis or discontinuing dialysis***, topics previously discussed. However, it should be emphasized that comprehensive conservative care must be offered regardless of these decisions. **Comprehensive conservative care** is intended for patients who are unlikely to benefit from dialysis and is based on reliable tools and patients who consciously choose this approach^87^.
- ***Palliative dialysis, incremental dialysis, and time-limited dialysis trials***, as previously discussed and described in Chart 2.
- ***End-of-life care:*** It refers to the care provided to patients in the final days or weeks of life, when clinical deterioration is irreversible and death is approaching. It involves comprehensive assessment, attention to religious/spiritual needs, active participation of an interdisciplinary team with expertise and skills in this area, and willingness to provide information to family members/caregivers regarding all upcoming steps. This care also includes support for family members/caregivers, the actual event of death, and respect for the grief experienced by their loved ones^83,88,89,90^.

Despite its benefits, the implementation of palliative care in nephrology still faces barriers in Brazil and worldwide, including misconceptions that equate it exclusively with end-of-life care, limited access to services, and insufficient training among nephrologists and multidisciplinary teams. The challenges include improving education and training in palliative care principles for professionals working in nephrology and developing integrated care models that incorporate palliative care into routine nephrology practice^84,89,90,91^.

### Brasilian Society of Nephrology (BSN) Recommendations on How to Conduct The Process of Refusing or Discontinuing Dialysis

**Figure 4:**
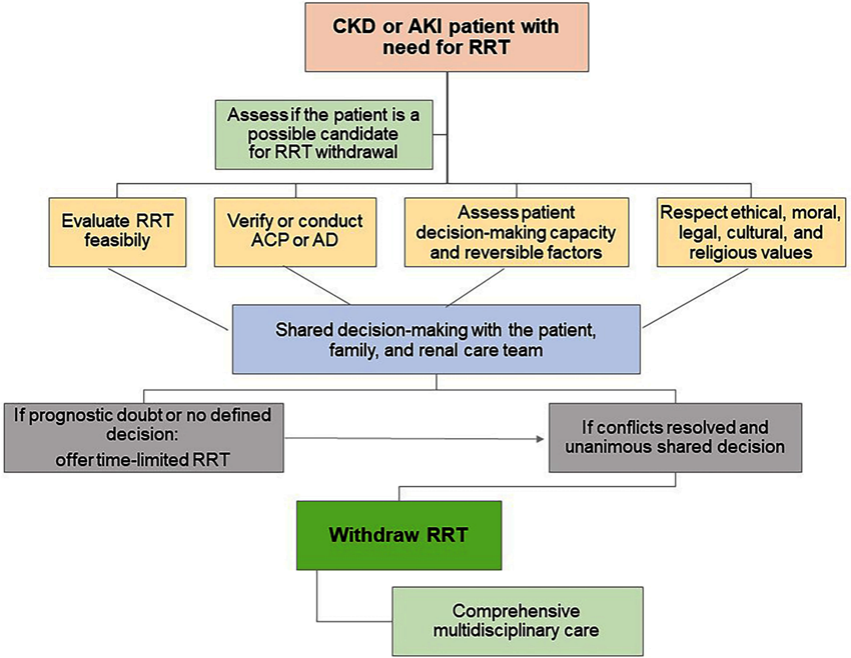
Flowchart of the steps for discontinuing or refusing Renal Replacement Therapy.

The BSN presents guidelines for conducting the dialysis withdrawal process in patients with renal failure, whether due to CKD or AKI, which are described in Chart 4 and Figure 4.

**Chart 4:** Guidelines from the Brazilian society of nephrology to nephrologists on how to conduct the process of dialysis refusal or discontinuation^8,15,32,48,55,56,57,58,80^

Steps	How to do it
Identify eligible patients	– See Chart 3. – Attention should be given to patients who are incapable of making decisions for clinical, psychiatric, or neurological reasons, in which case family members or legal representatives should be involved in the decision-making process. – Evaluate possible reversible causes and manage them before making a decision (biological, social, or psychological).
Conduct Advance Care Planning (ACP)	– See steps in Chart 1. – The ACP should be properly documented in the medical record, shared across the different levels of healthcare involved in the patient's care, and respected in its implementation.
Use some of the prognostic and quality of life assessment tools to support decision-making; results should be documented in medical records	– Charlson Comorbidity Index (available at: https://qxmd.com/calculate/calculator_879/charlson-comorbidity-index-score-cci-score). – Surprise question (“Would you be surprised if this patient died within the next 12 months?”). – Combined 6-month prognostic assessment criteria (available at: https://qxmd.com/calculate/calculator_135/6-month-mortality-on-hd). – Edmonton Frail Scale (available at: https://qxmd.com/calculate/calculator_595/edmonton-frail-scale). – The Australia-modified Karnofsky Performance Status (AKPS) scale46 (available at: https://www.spict.org.uk/wp-content/uploads/2020/11/Australia-modified-Karnofsky-Performance-Scale.pdf).
Respect the patient's wishes and encourage AD	– Encourage and guide patients to write their Advance Directive (AD) while in full possession of their mental faculties and capable of making their own decisions.
Provide therapeutic alternatives	– Present the patient with the available treatment options, including HD and PD. – Offer patients on PD or HD - with CKD or AKI - the possibility of palliative dialysis or a time-limited trial of dialysis as an alternative, especially in cases of limited life expectancy, impaired functionality, or the presence of severe comorbidities. – Consider incremental dialysis for patients with significant residual renal function (RRF), with continuous and thorough assessments to identify potential further loss of RRF and to adjust the dialysis dose accordingly.
Aligning decisions with bioethical and legal principles	– This position statement recommends that all hospitals and clinics performing RRT in patients with AKI or CKD establish a Bioethics Committee or Commission, as recommended by CFM Resolution No. 8/2015, which establishes the creation, functioning, and participation of physicians in Bioethics Committees78. – The Brazilian Federal Council of Medicine provides a Model Bylaws for this purpose, which may assist in its implementation^92^
Decisions should be shared and consensual	– The decision should be shared among the patient, physician, family members, and at least one additional member of the renal care team (psychology, nursing, social work). – When conflicts and disagreements arise, they should be resolved prior to decision-making.
Allow time for reflection	– Ensure at least two appointments at intervals agreed upon by the patient and their family members, always documented in the medical record.
Ensure knowledge of the risks and benefits of the decision to discontinue dialysis, advising that this decision may be reversible	– Explain the risks and benefits of different treatment options. – Provide information on invasive and artificial life-sustaining measures.
Record of decision-making	– Always document the decision in the medical record, so that the entire care team has access to what has been discussed.
Comprehensive care after the decision to withdraw dialysis treatment	– End-of-life care, provided by a trained multidisciplinary team, is recommended for all patients, their families, and caregivers experiencing the end-of-life process, regardless of whether they are on dialysis or comprehensive conservative care. – Ensure full access to management of symptoms (physical, psychological, social, and spiritual) expected in the context of end-stage kidney disease without dialysis support. – Ensure early outpatient consultation with a nephrologist and a multidisciplinary team, including palliative care team whenever possible, and guarantee access to long-term care hospitals.

Abbreviations – RRF: Residual Renal Function; AKI: Acute Kidney Injury; CKD: Chronic Kidney Disease; HD: Hemodialysis; PD: Peritoneal Dialysis; ACP: Advance Care Planning.

Abbreviations – CKD: Chronic Kidney Disease; AKI: Acute Kidney Injury; RRT: Renal Replacement Therapy; ACP: Advance Care Planning; AD: Advance Directive.

## Conclusion

Whether referring to discontinuation or non-initiation of dialysis (refusal), the decision must involve a shared discussion that respects patient autonomy and balances the principles of beneficence, nonmaleficence, and justice, as well as the patient’s clinical condition and the bioethical and legal aspects involved. The assessment of eligibility should consider the exclusion of potentially reversible clinical, psychological, and social factors. In addition, the healthcare team should adopt a supportive and evaluative approach to these situations, ensuring that the entire process is properly documented in the patient’s medical record.

## Data Availability

The entire dataset supporting the results of this study is available upon request to the corresponding author.
