# Supplementary material for: Position statement of the Brazilian Society of Nephrology on the refusal and discontinuation of dialysis
Source: J Bras Nefrol. 2025 Aug 15;47(3):e20250057. doi: 10.1590/2175-8239-JBN-2025-0057en (PMC12360398; doi:10.1590/2175-8239-JBN-2025-0057en)
Supplement: Translation

# Translation: Posicionamento da Sociedade Brasileira de Nefrologia sobre a recusa e a descontinuação da diálise

**DOI:** 10.1590/2175-8239-JBN-2025-0057pt

**Authors:** Dirceu Reis da Silva, Fernanda Salomão Gorayeb-Polacchini, Ana Flávia Moura, Cibele Isaac Saad Rodrigues, Maurício Younes-Ibrahim, Eduardo Rocha, Marina Aline Occhiena de Oliveira Neiva, Pedro Túlio Rocha, Patrícia Ferreira Abreu, José A. Moura-Neto

**Affiliations:** 1 Sociedade Brasileira de Nefrologia, São Paulo, SP, Brasil.; 2 Hospital de Clínicas de Porto Alegre, Porto Alegre, RS, Brasil.; 3 Instituto de Doenças Renais, Porto Alegre, RS, Brasil.; 4 Hospital de Base da Faculdade de Medicina de São José do Rio Preto, São José do Rio Preto, SP, Brasil.; 5 Escola Bahiana de Medicina e Saúde Pública, Salvador, BA, Brasil.; 6 Pontifícia Universidade Católica, Faculdade de Ciências Médicas e da Saúde, Sorocaba, SP, Brasil.; 7 Hospital Santa Lucinda, Sorocaba, SP, Brasil.; 8 Universidade do Estado do Rio de Janeiro, Rio de Janeiro, RJ, Brasil.; 9 Pontifícia Universidade Católica do Rio de Janeiro, Rio de Janeiro, RJ, Brasil.; 10 Renalle Consultoria e Serviços Médicos, Petrópolis, RJ, Brasil.; 11 Universidade Federal do Rio de Janeiro, Rio de Janeiro, RJ, Brasil.; 12 Hospital Santa Casa de Misericórdia de Goiânia, Goiânia, GO, Brasil.; 13 Hospital do Rim, Goiânia, GO, Brasil.; 14 Hospital São Lucas Copacabana, Rio de Janeiro, RJ, Brasil.; 15 Universidade Federal de São Paulo, São Paulo, SP, Brasil.

**Keywords:** Diálise, Injúria Renal Aguda, Diretivas Antecipadas, Suspensão de Tratamento, Obstinação Terapêutica, Cuidados Paliativos, Bioética

## Abstract

A falência renal é considerada uma condição limitante à vida, frequentemente exigindo Terapia Renal Substitutiva, como a diálise ou o transplante renal. A diálise pode ser eficaz no alívio dos sintomas e no prolongamento da vida, mas sua renúncia pode resultar em complicações graves e óbito. A decisão sobre sua descontinuação ou a recusa à diálise deve ser tomada de forma compartilhada entre paciente, familiares e equipe de saúde, considerando a condição clínica, a qualidade de vida e as preferências do paciente. Essa decisão, que envolve aspectos clínicos e bioético-legais, é complexa e exige uma compreensão coletiva do processo. A renúncia à diálise representa um desafio para nefrologistas e equipes de saúde, devido à ausência de diretrizes claras, o que pode comprometer a segurança do processo e a dignidade do paciente. Neste posicionamento, a Sociedade Brasileira de Nefrologia recomenda que tanto a recusa quanto a descontinuação da diálise envolvam a identificação de pacientes elegíveis, a aplicação de ferramentas de avaliação prognóstica, a tomada de decisão compartilhada, o planejamento antecipado do cuidado e a oferta de alternativas de diálise. A decisão deve ser consensual, com tempo adequado para reflexão, e o serviço de saúde deve oferecer manejo abrangente dos sintomas físicos, psicológicos, sociais e espirituais, além de cuidados de fim de vida. A documentação adequada em prontuário é fundamental para garantir a transparência do processo. Assim, a renúncia à diálise deve ser uma decisão informada, que respeite a autonomia do indivíduo e equilibre considerações clínicas, bioéticas, espirituais e legais.

## Introdução

Especialmente em seus estágios mais avançados, a falência renal pode ser considerada uma condição potencialmente limitante à vida, sendo então necessária a adoção de uma Terapia Renal Substitutiva (TRS), quer seja uma das modalidades de diálise, quer seja o transplante renal^1^. No escopo da presente discussão, abordamos de forma abrangente os cenários de doença renal crônica (DRC) e injúria renal aguda (IRA), e nos referimos à diálise como conceito genérico, englobando as suas mais variadas alternativas, como a diálise peritoneal (DP), a hemodiálise (HD) e a hemodiafiltração (HDF).

A diálise é amplamente reconhecida como um tratamento eficaz para aliviar a sintomatologia decorrente da falência renal avançada e para prolongar a vida desses pacientes^1^. Por se tratar de uma terapia de suporte à vida, sua descontinuação ou recusa resulta, inexoravelmente, em complicações graves, como hipervolemia, hiperpotassemia ou uremia, com tendência a desfecho fatal em aproximadamente 7,8 dias^2^.

Portanto, considerando a condição clínica, a qualidade de vida e as preferências do paciente, a decisão pela renúncia ao tratamento dialítico deve ser tomada de forma compartilhada entre o doente, sua família e a equipe de saúde^3^. Trata-se de um julgamento complexo (ver Figura 1), que demanda um processo coletivo de compreensão e construção. Nesse contexto, a renúncia à diálise representa um desafio para os médicos nefrologistas e para a equipe de cuidados, resultando em limitações na consistência da deliberação e na disponibilização dos cuidados de fim de vida^4^.

**Figura 1:** Aspectos que podem interferir no processo de descontinuação ou recusa à diálise.

Abreviaturas – DRC: Doença Renal Crônica; IRA: Injúria Renal Aguda.

No Brasil, ainda se fazem necessárias a adequada capacitação dos profissionais de saúde e a elaboração de diretrizes nacionais, em busca de maior segurança na definição das condutas, preservação da dignidade dos pacientes, proteção emocional de familiares e profissionais de saúde, além de menor risco jurídico^5^.

Os objetivos deste documento são: 1) contribuir para a qualidade do processo de morte de pacientes com doença renal avançada; 2) minimizar os efeitos adversos dessa experiência dolorosa para as famílias e colaborar para o conforto dos profissionais de saúde nesse contexto; e 3) servir como base para a elaboração de protocolos e instrumentos destinados a profissionais, administradores hospitalares e formuladores de políticas de saúde, em conformidade com a realidade local.

## Discussão

### Definição

Apesar de sua crescente aceitação em países desenvolvidos, a decisão de suspender a oferta de diálise no contexto de pacientes com falência renal ainda carece de uma definição clara e consistente entre estudos^4–7^. No presente posicionamento, tratamos das suas duas formas possíveis: a recusa e a descontinuação. A recusa, mais comum, significa a decisão de não iniciar o tratamento dialítico em pacientes com doença renal avançada, nos quais a diálise estaria tradicional e tecnicamente indicada como intervenção de suporte à vida^7^. Por outro lado, a descontinuação consiste na decisão de interromper a diálise já iniciada e necessária para o suporte à vida^6–7^, ou na constatação de óbito adequadamente atribuído à falência renal em paciente que tenha interrompido a diálise^4^.

Neste documento, adotamos o termo “renúncia à diálise” como um conceito abrangente, que contempla duas possibilidades distintas: a recusa e a descontinuação do tratamento dialítico.

### Epidemiologia

A renúncia à diálise tem ocorrido com frequência crescente na população com falência renal, apresentando ampla variabilidade geográfica. Essa variação está relacionada ao perfil dos pacientes, à influência de crenças culturais, religiosas e espirituais, a valores éticos, bem como à maior ou menor disponibilidade de TRS^4,8^.

Em uma revisão sistemática que avaliou a sobrevida de pacientes com DRC avançada e indicação de TRS, mas que optaram pelo tratamento conservador (configurando recusa à diálise), essa decisão resultou em uma mediana de sobrevida de pelo menos seis meses, variando entre 6,3 e 23,4 meses^9^. Em outra revisão sistemática e metanálise, que analisou pacientes idosos com DRC avançada, a sobrevida em um ano foi semelhante entre os tratados com diálise e os que seguiram com tratamento conservador, embora não tenham sido realizadas comparações de qualidade de vida^10^.

Em um estudo do DOPPS, envolvendo 259.343 pacientes, 12% dos óbitos foram atribuídos à descontinuação da diálise. Nesses casos, observou-se uma sobrevida com tempo mediano de sete dias (intervalo interquartil de 5 a 9 dias) após a interrupção do tratamento, sendo que apenas 4,4% morreram após mais de 30 dias^11^. A descontinuação da diálise tende a ser mais frequente entre idosos^12^, pacientes do sexo feminino^13^, residentes em áreas rurais^13^, com doenças degenerativas^12^ ou incapacidades^13^, pacientes em DP intermitente^12^ ou institucionalizados^12^.

Em países com alta renda *per capita*, a descontinuação da diálise tornou-se uma das principais causas de óbito entre pacientes em terapia dialítica^11–18^. Em que pese a escassez de dados brasileiros na literatura, presume-se que essas taxas sejam inferiores às observadas em países desenvolvidos, por razões sociais, culturais, religiosas e econômicas^5^.

### Pacientes Com DRC em Estágio Avançado em Tratamento Conservador ou em Clínicas de Diálise

Por um lado, enquanto o transplante renal possui critérios de elegibilidade e contraindicações que não permitem sua oferta universal aos pacientes com DRC em estágio avançado^19^, a diálise é virtualmente aplicável a qualquer indivíduo que necessite de TRS. Além disso, as mudanças demográficas em curso no país – em especial o envelhecimento populacional e o aumento de pessoas com comorbidades crônicas – justificam, em grande parte, o crescimento da prevalência de casos de DRC avançada^20^. Portanto, torna-se crucial discutir com pacientes com DRC avançada e seus familiares o prognóstico, considerando suas condições médicas, as possíveis opções terapêuticas (TRS ou tratamento conservador), com suas respectivas vantagens e desvantagens, bem como a necessidade do Planejamento Antecipado de Cuidados (PAC), de forma a proporcionar conforto, paz e dignidade no fim de vida^8,21^. As etapas para a realização do PAC estão descritas no Quadro 1.

**Quadro 1:** Etapas do planejamento antecipado de cuidados (PAC)^22^

Etapas	Objetivos
Preparação	– Certificar-se dos detalhes do diagnóstico, do prognóstico e das opções de tratamento disponíveis antes da conversa com o paciente.
Permissão/checar autonomia	– Avaliar inicialmente se o paciente pode tomar a decisão sozinho e se há a necessidade da presença de familiares. – O paciente, avaliado como competente, deve ser questionado sobre o desejo de protagonizar a tomada de decisão ou se prefere compartilhá-la com um familiar ou ente próximo de sua confiança. – Pacientes com sintomas descompensados devem ter esse diálogo somente após melhora dos sintomas.
Identificação da percepção do paciente sobre a doença, seus valores e desejos	– Resgatar, por meio de escuta ativa, a compreensão do paciente e de seus familiares sobre diagnóstico, prognóstico e alternativas usuais de tratamento.
Conversar sobre possíveis dilemas e preferências de cuidados	– Explicar os riscos e benefícios das diferentes opções de tratamento. – Esclarecer sobre medidas invasivas e artificiais de suporte de vida.
Tomada de decisão e plano de cuidados	– Traçar o plano de cuidados, alinhando as opções terapêuticas disponíveis às preferências e prioridades do paciente.
Documentar as decisões	– Sempre registrar as decisões em prontuário, para que toda a equipe responsável pelo paciente tenha acesso ao que foi discutido e acordado. – Incentivar o paciente a redigir sua Diretiva Antecipada de Vontade (DAV)*.
Identificar um procurador de cuidados de saúde do paciente	– Incentivar o paciente a selecionar uma pessoa de sua confiança para representá-lo em situações em que ele(a) não possa se expressar ou tenha sua autonomia comprometida. – Caso o paciente não tenha indicado um representante, é recomendável incentivá-lo a compartilhar suas preferências com pessoas próximas e de confiança, de modo a garantir que suas vontades sejam respeitadas.
Revisar o PAC	– Validar o que foi conversado e oferecer ao paciente a possibilidade de revisão, a qualquer momento, do plano de cuidados.

Abreviaturas – PAC: Planejamento Antecipado de Cuidados.

Notas – *Elaboração de uma DAV na forma de testamento vital e/ou mandato duradouro. Ver detalhamento nos aspectos (bio)éticos.

As principais razões para a descontinuação da diálise nessa população incluem fatores clínicos (comorbidades crônicas debilitantes, incapacidade, fragilidade, complicações agudas e dificuldades de acesso) e fatores psicossociais (carga de saúde mental, falta de apoio social e razões logísticas)^2,4,13^.

Com o objetivo de reduzir a carga do tratamento e otimizar a qualidade de vida, devem ser oferecidas aos pacientes alternativas aos regimes tradicionais de diálise^3–4,21^ (diálise incremental^22–26^, diálise paliativa^27–28^ ou diálise por tempo limitado^29–31^), conforme detalhado no Quadro 2 ^22–31^.

**Quadro 2:** Alternativas de diálise (hemodiálise ou peritoneal) em pacientes com desejo ou candidatos à descontinuação ou recusa da diálise

Alternativas de Diálise	Tipo de doença renal	Como fazer	Objetivo	Acompanhamento
Diálise incremental^22–26^	DRC	– Pacientes com FRR; – Geralmente 2 a 3 horas, 2 vezes por semana.	– Preservar a FRR, menor custo e taxa de hospitalização, melhor qualidade de vida e adesão ao tratamento.	– Avaliar perda da FRR; – Ajustar, se necessário, o tempo ou frequência conforme quadro clínico laboratorial do paciente.
Diálise paliativa^27,28^	DRC ou IRA	– Pacientes com expectativa de vida limitada ou comorbidades graves; – Diálise ou UF, apenas se sintomas.	– Conforto, melhor qualidade de vida, controle dos sintomas.	– Acompanhamento do paciente com foco no controle de sintomas.
Diálise por tempo limitado^29–31^	DRC ou IRA	– Tempo limitado acordado com o paciente e familiares; – Estabelecer objetivo do tratamento temporário.	– Tempo limitado de tratamento devido à dúvida prognóstica, conflitos não resolvidos, causas potencialmente reversíveis.	– Avaliação do paciente ao final do período, de acordo com os objetivos propostos; – Caso necessário, reavaliar a conduta durante o período (mudança de decisão, quadro clínico ou prognóstico).

Abreviaturas – HD: Hemodiálise; DP: Diálise Peritoneal; DRC: Doença Renal Crônica; IRA: Injúria Renal Aguda; FRR: Função Renal Residual; UF: Ultrafiltração.

Apesar de a descontinuação da diálise ser frequente em países de alta renda, as abordagens clínicas atualmente adotadas nas unidades de diálise costumam ainda não atender às necessidades de pacientes em fase final de vida. Em um estudo canadense conduzido em unidades de diálise, pacientes relataram pouco conhecimento sobre a trajetória de sua doença e sobre as opções de cuidados paliativos; entre eles, 61% disseram se arrepender da decisão de iniciar diálise, 27% disseram preferir morrer hospitalizados e menos de 10% relataram ter tido a oportunidade de discutir questões relacionadas aos cuidados de fim de vida com seu nefrologista nos últimos 12 meses^32^. Sendo assim, os centros de diálise precisam desenvolver suas equipes e disseminar orientações escritas sobre como e quando ofertar a descontinuação da diálise, e sobre como os pacientes devem prosseguir sob cuidados de saúde integral^8^. Além disso, é fundamental incorporar treinamento em Cuidados Paliativos para médicos residentes e estagiários de Nefrologia, com valorização do manejo de sintomas e do estabelecimento do PAC^33^.

Para pacientes em tratamento dialítico em fase final de vida, recomenda-se discutir os cuidados apropriados de terminalidade e a eventual descontinuação da diálise por meio do PAC. Por outro lado, ao se avaliar a decisão de descontinuidade em pacientes com perspectiva indefinida de proximidade com a morte, devem ser investigados eventuais fatores potencialmente tratáveis ou manejáveis, como: depressão, ansiedade e dor; insatisfação ou desconforto com a diálise; apoio social inadequado; e preocupação com a percepção de se tornarem um fardo para os entes queridos^4,21^.

Na condução compartilhada da decisão de não iniciar (recusa) ou de descontinuar a diálise do paciente com DRC, devem ser oferecidos cuidados clínicos e multidisciplinares abrangentes, com uma perspectiva holística centrada no paciente, que inclua o manejo ativo dos sintomas, bem como suporte psicológico, social, familiar e espiritual^21^.

### Aspectos Específicos da Recusa À Diálise em Pacientes Com Ira ou em Leito de Unidade de Terapia Intensiva (UTI)

A IRA é um evento frequente, podendo atingir até 57,3% dos pacientes em leitos de UTI^34^, e cuja mortalidade varia inversamente aos seus estágios de classificação propostos pelo *Kidney Disease: Improving Global Outcomes* (KDIGO)^34,35^. Trata-se de uma síndrome clínica com fenótipos variáveis, que pode acometer pacientes de todas as faixas etárias, sendo mais frequente na população idosa^36^.

A tomada de decisão sobre o não início (recusa) ou a descontinuação da TRS em pacientes com IRA pode ser mais complicada do que em pacientes com DRC, devido aos seguintes fatores^37^: (a) potencial de recuperação da função renal; (b) mesmo em casos graves, a doença aguda pode ser percebida por pacientes e médicos como reversível^38^; (c) pacientes gravemente doentes frequentemente estão incapacitados de tomar decisões médicas; (d) poucos pacientes gravemente enfermos possuem PAC e/ou DAV, e, mesmo quando existentes, essas ferramentas não parecem auxiliar no processo de tomada de decisão por seus substitutos^39^. Os contextos clínicos de pacientes elegíveis para a renúncia à diálise em casos de IRA^37,39^ não diferem essencialmente daqueles observados em pacientes com DRC, estando descritos no Quadro 3.

**Quadro 3:** Pacientes potencialmente candidatos à recusa ou descontinuação da diálise

1.	Pacientes com capacidade preservada de decisão, plenamente informados, que optem pela renúncia à diálise de forma voluntária^48^
2.	Pacientes sem capacidade decisória (causas clínicas, neurológicas ou psiquiátricas) e que tenham PAC^33,53^ documentada, ou cujos agentes legais devidamente nomeados optem pela renúncia à diálise^48^
3.	Pacientes com dano neurológico irreversível e profundo, com ausência de sinais de pensamento, sensação, comportamento intencional e consciência de si e do ambiente^48^
4.	Pacientes com DRC avançada de causa não renal ou cuja condição médica impossibilite, do ponto de vista técnico, o procedimento de diálise^48^
5.	Pacientes com DRC avançada sem diálise, com alto risco de óbito no próximo ano^32^ (considerar o uso de uma avaliação de predição de mortalidade em 6 meses combinada^54^ ou a “Pergunta Surpresa” (“Você ficaria surpreso se este paciente viesse a óbito nos próximos 12 meses?”)^55^ e avaliação de sintomas, como a Escala de Avaliação de Sintomas de Edmonton^56^)
6.	Pacientes em que a diálise sustenta a vida em condições comórbidas nas quais a qualidade de vida e a experiência do viver são inadequadas e indesejadas, ou em que o desfecho fatal é assegurado (por exemplo, um paciente com câncer avançado, sem proposta terapêutica, ou sepse com falência de múltiplos órgãos)^57^
7.	Pacientes em que o esforço para implementação da diálise, bem como suas complicações, superam os benefícios de prolongamento da vida (por exemplo, paciente com fragilidade progressiva, que está ficando acamado, ou com deficiência cognitiva grave)^57^

Abreviaturas – PAC: Planejamento Antecipado de Cuidados; DRC: Doença Renal Crônica.

Escores de avaliação de sobrevida do paciente grave podem auxiliar no processo de tomada de decisão quanto à renúncia à diálise^40–44^, sobretudo em idosos > 75 anos^44^. Por meio de ferramentas específicas, é possível configurar critérios objetivos traduzidos por índices que revelam alto grau de comorbidades (ex: Índice Modificado de Comorbidades de Charlson >8^45^) ou de comprometimento de funcionalidade (ex: AKPS ≤ 40%^46^).

Em pacientes graves com IRA associada à falência de múltiplos órgãos e/ou decorrente de outras comorbidades descompensadas, a renúncia à diálise passa a ser considerada quando os métodos dialíticos empregados deixam de ter papel terapêutico e passam apenas a postergar o desfecho final. Nesses pacientes, a decisão de não início ou descontinuação da TRS, com o intuito de instituir cuidados proporcionais, é um dos desafios da prática nefrológica hospitalar, ainda que a disponibilidade de novas tecnologias possibilite a oferta de TRS para a maioria dos pacientes^47^. Por se tratar de uma questão complexa, não existe uma única abordagem que possa ser adequada a todos os casos, sendo essencial que as decisões sejam sempre tomadas de forma individualizada^37,48^.

Além disso, discutir com o paciente ou seus familiares sobre o prognóstico de longo prazo é importante para garantir que seus desejos sejam respeitados. Dados de pacientes com internações prolongadas em leito de UTI (2 semanas) demonstraram que apenas 38% haviam discutido com seus médicos sobre o prognóstico, e que 47% dos que preferiram uma abordagem paliativa acreditavam estar recebendo um tratamento contrário^49^. A oferta de diálise por tempo limitado^29,30^ ou a diálise paliativa^27^ pode ser uma opção clínica e eticamente apropriada^30^ para pacientes com IRA, conforme resumido no Quadro 2.

Uma vez tomada a decisão de limitar medidas invasivas, é importante ressaltar que a descontinuação do suporte de vida pode ocorrer de forma gradual. A descontinuação da diálise é, provavelmente, uma das primeiras medidas adotadas, enquanto a descontinuação da alimentação, hidratação e ventilação mecânica tendem a ser as últimas intervenções retiradas^50^. O acompanhamento por equipe de Cuidados Paliativos deve ser considerado para auxiliar no gerenciamento dos sintomas de difícil controle, bem como na resolução de eventuais conflitos entre paciente, familiares e profissionais de saúde. As famílias devem estar cientes da possibilidade de que os pacientes se recuperem da IRA. A transferência para um andar fora da UTI deve ser considerada, a depender das preferências do paciente, da capacidade de enfrentamento dos familiares e da existência de equipe treinada para cuidados de fim de vida^37^.

Diante da complexidade da questão, nenhum nível de evidência foi suficiente para permitir recomendações formais em diferentes pontos^51^. Com o crescente desenvolvimento de ferramentas de inteligência artificial, não será surpreendente se, em um curto prazo, a validação de novos modelos algorítmicos vier a auxiliar categoricamente nas decisões relacionadas aos cuidados de fim de vida em pacientes graves^52^.

### Pacientes Elegíveis Para o Processo de Renúncia À Diálise

Diferentes diretrizes e recomendações^8,32,48^ foram publicadas para orientar as situações em que os pacientes são potencialmente elegíveis para a renúncia à diálise, e estão compiladas no Quadro 3 ^33,48,53–57^.

Dentre as diferentes diretrizes, a *Renal Physicians Association* (RPA) e a *American Society of Nephrology* (ASN) reuniram-se com representantes de diversas organizações da comunidade de diálise - pacientes renais, familiares, médicos internistas, bioeticistas e especialistas em políticas públicas - e publicaram o *Clinical Practice Guideline on Shared Decision-Making in the Appropriate Initiation of and Withdrawal from Dialysis*, onde são estabelecidas nove recomendações sobre a renúncia à diálise, incluindo os pacientes candidatos ao processo^48^.

Por sua vez, o KDIGO publicou que a renúncia à diálise é ética e clinicamente aceitável, devendo-se sempre abordar fatores potencialmente tratáveis que eventualmente contribuam para essa decisão (como depressão e outras enfermidades mentais), ou outros sintomas (como dor, prurido, síndrome das pernas inquietas, distúrbios do sono e fadiga), além de considerar fatores sociais reversíveis^8^.

A renúncia à diálise representa um desafio particular em pacientes acometidos por demência, uma vez que esses indivíduos são incapazes de tomar suas próprias decisões. Um estudo com dados de sete países reconheceu que a demência está associada a um risco aumentado de óbito e a uma probabilidade duas vezes maior de descontinuação da diálise, reforçando a importância do rastreamento médico adequado e regular do comprometimento cognitivo dos pacientes^58^.

### Aspectos (Bio)Éticos e Legais Que Tangenciam A Recusa ou A Descontinuação da Diálise

- “*The good physician treats the disease; the great physician treats the patient who has the disease*.” – Sir William Osler

Com os avanços nas TRS, a sobrevida dos pacientes tem aumentado significativamente, especialmente entre aqueles com DRC^59^. No entanto, não basta dispor de bons recursos tecnológicos e equipes multiprofissionais treinadas; é fundamental que o paciente esteja no centro da nossa atenção e dos cuidados prestados^60^. Assim, ao se considerar o indivíduo sob uma perspectiva biopsicossocial, fundamentada na bioética principialista, é essencial considerar os princípios da beneficência (fazer o bem) e da não maleficência (não fazer o mal), não apenas em relação aos pacientes, mas também à sua rede de apoio - que inclui familiares, cuidadores e a equipe multiprofissional^61^.

Princípios igualmente basilares no olhar humanizado a pacientes que necessitam de TRS incluem a autonomia e o protagonismo dos indivíduos, a corresponsabilidade entre os profissionais da saúde e os pacientes, a valorização de suas vulnerabilidades, a criação de vínculos solidários e a participação coletiva no processo de gestão decisória^62^.

Atualmente, existe um arsenal para a manutenção de variáveis vitais, que oferece aos profissionais de saúde a possibilidade de adiar, por longos períodos, o processo natural de morrer. Cabe, entretanto, questionar: qual é o impacto dessa nova realidade na qualidade de vida dos pacientes? O complexo tanatológico de lutar contra a morte e a obsessão por manter a vida biológica a qualquer custo têm conduzido ao conceito de obstinação terapêutica, ou distanásia, que contraria os princípios da dignidade humana diante dos limites da medicina e da ciência^63,64^. Esse tipo de abordagem, que prioriza a quantidade em detrimento da qualidade de vida, deve ser considerada uma má prática clínica, além de configurar uma postura antiética, prevista como infração no Código de Ética Médica^65^.

Desde 2006, o Conselho Federal de Medicina (CFM), por meio da Resolução nº 1.805/2006, passou a permitir aos médicos a não implementação e a suspensão de tratamentos que prolonguem a vida de pacientes em fase terminal de doença grave e incurável, desde que respeitada a vontade do paciente ou de seu representante legal^66^. As principais justificativas do CFM para essa resolução encontram respaldo nos artigos 1^o^ e 5^o^ da Constituição Federal Brasileira, que estabelecem o princípio da dignidade da pessoa humana como um dos pilares fundamentais do país e proíbem a submissão de qualquer indivíduo à tortura ou a tratamentos desumanos ou degradantes. Essa resolução está em consonância com o posicionamento de entidades internacionais, como a Associação Médica Mundial, o Conselho Europeu, a Corte Europeia de Direitos Humanos (CEDH) e a Organização das Nações Unidas para a Educação, a Ciência e a Cultura (UNESCO).

Ainda não temos respostas definitivas para o que pode ocorrer entre o “fazer tudo” e o “não fazer nada”. Condutas mais ou menos invasivas devem respeitar a individualidade e a biografia de cada paciente, sendo guiadas pelos princípios bioéticos da autonomia, justiça, beneficência e não maleficência, visando conforto e qualidade de vida. O objetivo deve ser, sempre, cuidar dos pacientes de uma maneira que seja consistente com seus valores, em um momento de incomparável vulnerabilidade, quando raramente conseguem falar por si mesmos^67^. Assegurar que o fim da vida ocorra no tempo certo, com dignidade, cuidado e o máximo de conforto possível é o que se denomina ortotanásia (“boa morte”)^65,68^.

Um conceito mais atual é o da calotanásia ou kalotanásia, ainda pouco difundido, mas mais abrangente do que a ortotanásia. Refere-se ao revivalismo da morte, ao uso racional de recursos terapêuticos para o controle da dor e do sofrimento, evitando assim o prolongamento desnecessário do processo de morrer. Esse conceito tem fundamento no movimento *hospice*, que se baseia no cuidado respeitoso às crenças e valores do paciente e de sua família^69^.

Ressalte-se ainda que a eutanásia – entendida como a morte por misericórdia ou piedade – é considerada crime no território brasileiro. Essa prática pressupõe uma “ação” que causa a morte, seja de forma ativa, seja de forma passiva, sendo que, neste último caso, configura-se por negligência^65,68^.

Adicionalmente, é essencial reconhecer que a descontinuação e a não implementação de intervenções de suporte de vida no contexto de uma doença terminal diferem do conceito de eutanásia. A *International Association for Hospice and Palliative Care (IAHPC)* e a *European Association for Palliative Care (EAPC)* publicaram posicionamentos acerca da questão da eutanásia^70,71^, definindo-a como a ação de um médico ao administrar medicamentos a um paciente com o intuito de causar sua morte, com base em uma solicitação voluntária feita pelo próprio paciente em plena capacidade de decisão.

A crise humanitária da diálise, denunciada pela Sociedade Brasileira de Nefrologia (SBN), se encaixa perfeitamente na definição mais recente de morte: a mistanásia^68^, sendo “mis” um prefixo que significa infeliz. Essa é a nomenclatura utilizada para se referir à morte de pessoas, muitas vezes socialmente excluídas, sem qualquer assistência em saúde ou com atendimento precarizado. O diagnóstico tardio – ou ausente – de DRC, a escassez de unidades de diálise suficientes para atender à demanda de pacientes com DRC e IRA, e a ausência de recursos para suporte de TRS durante a pandemia de COVID-19 são exemplos desse tipo de morte no contexto da Nefrologia^72–74^.

As Diretivas Antecipadas de Vontade (DAV) são documentos que expressam o conjunto de desejos previamente e expressamente manifestados pelo paciente sobre os cuidados e tratamentos que deseja ou não receber quando estiver incapacitado de expressar sua vontade de forma livre e autônoma (Resolução CFM 1995/2012, Art. 1º)^75^. As DAV podem ser formalizadas por meio de dois instrumentos distintos: o testamento vital e o mandato duradouro. Ambos podem coexistir e serão utilizados quando o paciente não puder se expressar livre e conscientemente. O mandato duradouro possibilita que o paciente nomeie um ou mais procuradores, a serem consultados na eventualidade de incapacidade temporária ou definitiva do paciente para tomar decisões sobre tratamentos ou procedimentos. Esse instrumento pode ser utilizado quando o doente não manifestou previamente sua vontade ou em situações em que há condições não previstas (lacunas), cabendo ao procurador decidir, sempre ancorado na vontade presumida do paciente^76^.

O outro tipo de DAV, além do mandato duradouro, é o chamado testamento vital. Trata-se de um documento jurídico que veio na esteira do “*living will*”, instituído nos EUA desde a década de 1960. Este documento permite que os pacientes explicitem suas preferências quanto aos tratamentos que desejam autorizar, caso se encontrem em estado terminal, incluindo a possibilidade de suspender procedimentos extraordinários e considerados fúteis^75,76^.

De acordo com a Resolução CFM 1995/2012, o registro das DAV pode ser realizado pelo médico no prontuário do paciente, desde que autorizado por este ou por seu procurador^75^. Não são exigidas assinaturas de testemunhas, uma vez que o médico, em razão de sua profissão, possui fé pública, e seus atos têm validade legal e jurídica. Caso deseje, a pessoa pode registrar suas DAV em cartório, com testemunhas, mas não há essa obrigatoriedade^75,76^.

Nefrologistas frequentemente se veem envolvidos em questões intimamente relacionadas à vida e à morte de pacientes, tanto no contexto da DRC quanto da IRA, tendo que tomar decisões inquietantes. Essas decisões melhor se operacionalizam quando compartilhadas com membros de Comitês de Bioética dos hospitais, com a equipe multiprofissional das clínicas de diálise e, especialmente, com os próprios pacientes e seus familiares quando se opta por cuidados paliativos, ortotanásia e calotanásia. Para os nefrologistas, ter em mente os princípios bioéticos auxilia na não indicação ou mesmo na retirada da diálise, quando isso representar o melhor que se possa fazer pelo paciente, sempre com respeito à sua autonomia^77^.

A decisão de suspender ou de não indicar a diálise em pacientes com DRC não deve ser tomada de forma abrupta e deve implicar em um processo documentado com termo de consentimento livre e esclarecido assinado pelas partes envolvidas^78^. Trata-se sempre de uma decisão compartilhada entre a equipe responsável (médico e demais profissionais vinculados), a família e, sempre que possível, o próprio paciente, podendo incluir, se necessário, a consulta a especialistas em (bio)ética e/ou legislação. Essas situações devem ainda assegurar o cuidado, seja pela própria equipe nefrológica ou por meio de um Serviço de Cuidados Paliativos experiente e eficiente^79^.

O posicionamento publicado pelo Comitê de Bioética da Academia Nacional de Cuidados Paliativos inclui as seguintes diretrizes: (1) do ponto de vista da bioética, não existe distinção efetiva entre a descontinuação e a recusa de intervenções de suporte de vida; (2) não é ético iniciar ou manter medidas de suporte de vida em contextos nos quais estas estiverem em desacordo com os valores e objetivos de cuidado definidos em conjunto com os pacientes e seus familiares; (3) tratamentos fúteis – aqueles sem qualquer possibilidade de alcançar sequer seu objetivo fisiológico – não devem ser iniciados ou mantidos, mesmo a pedido do paciente ou de seus representantes; (4) com relação a tratamentos capazes de prolongar a vida de um paciente, sua manutenção pode ser inapropriada quando houver questionamentos quanto à sua base ética, e a sua recusa requer obrigatoriamente a pactuação de objetivos terapêuticos, considerando os valores e as perspectivas do paciente^80^.

Oferecer alternativas aos processos dialíticos que promovam melhor qualidade de vida é um dever dos profissionais de saúde. Além disso, é fundamental evitar a exposição dos pacientes a situações que possam causar sofrimento adicional, considerando o equilíbrio entre benefício e dano, entre o certo e o errado. Mesmo diante da terminalidade da vida, é imprescindível assegurar que esse processo ocorra com dignidade e respeito à condição humana.

### Cuidados Paliativos em Nefrologia

A definição de Cuidados Paliativos da Organização Mundial de Saúde (OMS) é a de uma abordagem integral que visa melhorar a qualidade de vida do paciente e de seus familiares, enfrentando de forma solidária os desafios associados a doenças que ameaçam a vida^81^, como é o caso da IRA ou da DRC avançada.

Deve-se realizar uma gestão eficaz do sofrimento, da angústia, dos sintomas físicos, emocionais, sociais, espirituais e do luto^82-84^. Os Cuidados Paliativos visam a satisfação do paciente e de seus familiares, o alívio da dor da perda, a redução de custos desnecessários e, em algumas situações muito específicas, o prolongamento da vida. Esses cuidados podem ser oferecidos em diferentes contextos, incluindo ambulatórios, hospitais, clínicas especializadas e no ambiente domiciliar^82–84^.

Os pilares dos Cuidados Paliativos estão descritos na Figura 2 ^82–84^.

**Figura 2:** Pilares dos Cuidados Paliativos.

Fonte – Adaptada das referências^82,83,84^.

Ao longo do tempo, o foco e os objetivos do cuidado vão progressivamente se deslocando desde uma ênfase em tratamentos modificadores da doença até tratamentos com intenção exclusivamente paliativa. O benefício dos Cuidados Paliativos aumenta à medida que cresce a carga de sintomas e a morte se impõe como sofrimento individual, ao longo de um *continuum* em que os tratamentos modificadores da doença perdem eficácia e aplicabilidade, estendendo-se até a fase ativa da morte e o luto dos entes próximos^85^. A participação potencial dos Cuidados Paliativos em relação ao momento da doença está representada na Figura 3.

**Figura 3:** Participação Potencial dos Cuidados Paliativos para os Pacientes em Relação ao Momento da Doença. A sobreposição entre os cuidados curativos e paliativos varia conforme a situação clínica.

Fonte – Adaptado da Comissão Permanente de Cuidados Paliativos da Sociedade Brasileira de Geriatria e Gerontologia. Vamos falar de Cuidados Paliativos, 2014, disponível em: https://sbgg.org.br/wp-content/uploads/2014/11/vamos-falar-de-cuidados-paliativos-vers-o-online.pdf.^85^

As linhas são representadas de forma reta apenas para fins didáticos, pois a intensidade de cada um dos tratamentos oferecidos simultaneamente oscila conforme as necessidades e prioridades do paciente e de sua família.

A taxa de mortalidade anual de pacientes em diálise é de 16,2%^20^, atingindo 38% entre aqueles com 75 anos ou mais e ultrapassando 50% em pacientes idosos frágeis^32^. Além disso, a trajetória da diálise é caracterizada por uma deterioração lenta, marcada por eventos agudos abruptos e somente parcialmente reversíveis, típica de falência orgânica^86^. Portanto, no planejamento do cuidado individualizado do paciente com IRA ou DRC, devem-se considerar as seguintes estratégias:

- ***Não iniciar a diálise ou descontinuar a diálise***, temas já discutidos anteriormente. Salienta-se, entretanto, a obrigatoriedade de se ofertar o tratamento conservador abrangente, independentemente dessas decisões. **O tratamento conservador abrangente** é aquele voltado para pacientes que não se beneficiariam da diálise e baseia-se em ferramentas fidedignas e em pacientes que optam conscientemente^87^.
- ***Diálise paliativa, diálise incremental e ensaio de diálise por tempo limitado***, já abordados anteriormente e descritos no Quadro 2.
- ***Cuidados de final da vida:*** Referem-se aos cuidados proporcionados aos pacientes nos últimos dias ou semanas de vida, quando a deterioração clínica é irreversível e a morte se aproxima. Envolvem avaliação abrangente, atendimento às necessidades religiosas/espirituais, participação ativa de uma equipe interdisciplinar com conhecimento e habilidade nessa área, além da disponibilidade para esclarecimentos aos familiares/cuidadores sobre todos os próximos passos. Esses cuidados incluem também o apoio aos familiares/cuidadores, o evento da morte propriamente dito e o respeito ao luto vivenciado por esses entes^83,88,89,90^.

Apesar dos benefícios, a implementação dos Cuidados Paliativos em Nefrologia ainda enfrenta barreiras no Brasil e no mundo, incluindo conceitos equivocados que os equiparam aos cuidados exclusivamente de fim de vida, acesso limitado aos serviços e formação insuficiente entre nefrologistas e equipes multiprofissionais. Os desafios envolvem melhorar a educação e o treinamento em princípios de Cuidados Paliativos para os profissionais que atuam na área de Nefrologia e desenvolver modelos de cuidados integrados que incorporem esses cuidados na prática rotineira da Nefrologia^84,89,90,91^.

### Orientações da Sociedade Brasileira de Nefrologia (Sbn) Sobre Como Realizar o Processo de Recusa ou de Descontinuação da Diálise

A SBN apresenta diretrizes para a condução do processo de renúncia à diálise em pacientes com falência renal, seja por DRC ou IRA, as quais estão descritas no Quadro 4 e na Figura 4.

**Quadro 4:** Orientações da sociedade brasileira de nefrologia aos nefrologistas sobre como conduzir o processo de recusa ou descontinuação da diálise^8,15,32,48,55–58,80^

Etapas	Como fazer
Identificar pacientes elegíveis	– Ver Quadro 3. – Atenção aos pacientes sem condição de tomada de decisão, por motivos clínicos, psiquiátricos ou neurológicos, quando familiares ou responsáveis legais devem estar envolvidos no processo de tomada de decisão. – Avaliar possíveis causas reversíveis e tratá-las antes da tomada de decisão (biológicas, sociais ou psíquicas).
Realizar o Planejamento Antecipado de Cuidados (PAC)	– Ver etapas no Quadro 1. – O PAC deve ser devidamente documentado no prontuário, compartilhado entre os diferentes níveis de atenção à saúde envolvidos no cuidado do paciente e respeitado em sua execução.
Usar algumas das ferramentas de avaliação prognóstica e de qualidade de vida para auxiliar na decisão; os resultados devem ser documentados em prontuário	– Índice de comorbidade de Charlson (disponível em: https://qxmd.com/calculate/calculator_879/charlson-comorbidity-index-score-cci-score). – Pergunta surpresa (“Você ficaria surpreso se este paciente viesse a óbito nos próximos 12 meses?”). – Critérios de avaliação prognóstica em 6 meses combinados (disponível em: https://qxmd.com/calculate/calculator_135/6-month-mortality-on-hd). – Escala de fragilidade de Edmonton (disponível em: https://qxmd.com/calculate/calculator_595/edmonton-frail-scale). – The Australia-modified Karnofsky Performance Scale (AKPS)46 (disponível em: https://www.spict.org.uk/wp-content/uploads/2020/11/Australia-modified-Karnofsky-Performance-Scale.pdf).
Respeitar o desejo do paciente e incentivar a DAV	– Incentivar e orientar o paciente a redigir sua Diretiva Antecipada de Vontade (DAV), em condições de pleno juízo e com capacidade de decidir sobre si mesmo.
Oferecer alternativas terapêuticas	– Apresentar ao paciente as opções de métodos terapêuticos disponíveis, incluindo HD e DP. – Oferecer, a pacientes em DP ou HD, com DRC ou IRA, a possibilidade de diálise paliativa ou de um período experimental de diálise como alternativa, especialmente em casos de expectativa de vida limitada, funcionalidade comprometida ou presença de comorbidades graves. – Considerar a diálise incremental para pacientes com função renal residual (FRR) significativa, com avaliações contínuas e criteriosas para identificar possíveis perdas adicionais de FRR e ajustar a dose de diálise conforme necessário.
Alinhar decisão com princípios bioéticos e legais	– Esse posicionamento recomenda que todos os hospitais e clínicas que realizam TRS em pacientes com IRA ou DRC constituam um Comitê ou Comissão de Bioética, nos moldes preconizados pela Resolução do CFM nº 8/2015, que estabelece a criação, o funcionamento e a participação dos médicos nos Comitês de Bioética^78^. – O Conselho Federal de Medicina disponibiliza um modelo de Regimento para essa finalidade que pode auxiliar na sua implementação^92^.
A decisão deve ser compartilhada e consensual	– A decisão deve ser compartilhada entre paciente, médico, familiares e, pelo menos, mais uma pessoa da equipe de cuidados renais (psicologia, enfermagem, assistência social). – Quando houver conflitos e discordâncias, esses devem ser resolvidas antes da tomada de decisão.
Permitir tempo para reflexão	– Garantir, no mínimo, dois encontros com o intervalo desejado pelo paciente e seus familiares, sempre documentados em prontuário.
Garantir conhecimento dos riscos e benefícios da decisão de interrupção da diálise, orientando que essa decisão pode ser reversível	– Explicar os riscos e benefícios das diferentes opções de tratamento. – Esclarecer sobre medidas invasivas e artificiais de suporte de vida.
Registro da tomada de decisão	– Sempre registrar a decisão em prontuário, para que toda a equipe responsável pelo paciente tenha acesso ao que foi discutido.
Cuidados abrangentes após a tomada de decisão de renúncia ao tratamento dialítico	– Recomenda-se cuidados de final de vida, realizado por equipe multiprofissional capacitada, para todos os pacientes, seus familiares e cuidadores que estejam vivenciando o processo de terminalidade da vida, independentemente de estarem em diálise ou em tratamento conservador abrangente. – Garantir o pleno acesso ao manejo dos sintomas (físicos, psicológicos, sociais e espirituais) esperados no contexto de terminalidade da doença renal avançada sem suporte de diálise. – Assegurar consulta precoce ambulatorial com nefrologista e equipe multiprofissional, sempre que possível com equipe de Cuidados Paliativos, e garantir acesso a hospital de retaguarda.

Abreviaturas – FRR: Função Renal Residual; IRA: Injúria Renal Aguda; DRC: Doença Renal Crônica; HD: Hemodiálise; DP: Diálise Peritoneal; PAC: Planejamento Antecipado de Cuidados.

**Figura 4:** Fluxograma das etapas para descontinuação ou recusa da Terapia Renal Substitutiva.

Abreviaturas – DRC: Doença Renal Crônica; IRA: Injúria Renal Aguda; TRS: Terapia Renal Substitutiva; PAC: Planejamento Antecipado de Cuidados; DAV: Diretrizes Antecipadas de Vontade.

## Conclusão

Quer refira-se à descontinuação ou ao não início da diálise (recusa), a decisão deve envolver uma discussão compartilhada, que respeite a autonomia do paciente e equilibre os princípios da beneficência, não maleficência e justiça, bem como as condições clínicas do paciente e os aspectos bioéticos e legais envolvidos. A avaliação de elegibilidade deve considerar a exclusão de fatores clínicos, psicológicos e sociais potencialmente reversíveis. Além disso, a equipe assistencial deve adotar um modelo de acolhimento e avaliação dessas situações, assegurando o registro adequado de todo o processo no prontuário do paciente.

## Disponibilidade de Dados

Todo o conjunto de dados que dá suporte aos resultados deste estudo está disponível mediante solicitação ao autor correspondente.
